# Breast mass imaging identification of rare pleomorphic adenoma after follow-up of gastric cancer surgery: a case report

**DOI:** 10.3389/fonc.2026.1759255

**Published:** 2026-02-11

**Authors:** XianJun He, LiLing Lu

**Affiliations:** Department of Radiology, Minzu Hospital of Guangxi Zhuang Autonom, Nanning, China

**Keywords:** breast tumor, diagnosis, identification, magnetic resonance imaging, pleomorphic adenoma

## Abstract

This study explores the imaging diagnosis and differential diagnosis of pleomorphic adenoma (PA) of the breast. PA is a rare benign tumor of the breast with a risk of recurrence, though it rarely undergoes malignant transformation. Therefore, extensive local excision is often performed, and a correct preoperative diagnosis of this lesion is important to ensure appropriate surgical treatment. Due to its rarity and nonspecific imaging findings, preoperative diagnosis is challenging and prone to misdiagnosis, especially in patients with a history of other malignancies. We present the case of a patient with a history of gastric cancer (GC) who presented with a breast mass that was pathologically confirmed to be PA. We analyzed the tumor’s imaging and pathological characteristics and discussed the diagnostic strategy.

## 1 Introduction

Pleomorphic adenoma (PA) is the most common benign tumor among salivary gland tumors. It most commonly occurs in the parotid gland. It is a tumor containing epithelial, myxoid, chondroid, and other tissues, with obvious pleomorphism and mixed shapes, and so it is called a pleomorphic adenoma or mixed tumor (1, 2). PA occurring in the breast is extremely rare. PA of the breast is classified as an epithelio-myoepithelial tumor, exhibiting unique histological features, including myoepithelial differentiation and a mucinous, cartilaginous-like matrix. Other benign breast tumors include adenomas, fibroepithelial tumors, hamartomas, papillary tumors, and more. Adenomas encompass tubular adenomas, lactating adenocarcinomas, and more. The more common fibroadenomas and phyllodes tumors fall under fibroepithelial tumors. Due to its rarity and tendency to be confused with fibroadenoma, phyllodes tumor, or mucinous carcinoma, PA of the breast can be challenging to distinguish on imaging studies, making preoperative diagnosis difficult. PA of the breast tends to occur in middle-aged and elderly women, with most reported cases occurring in individuals over 40 years of age, with the predilection site being in the subareolar area (3). However, there are few relevant imaging reports (4–8), and preoperative diagnosis is difficult. Therefore, this article aims to report a case of pathologically confirmed PA of the breast, focusing on its detailed imaging features on enhanced CT and MRI and combined with the patient’s history of gastric cancer (GC) to explore its differential diagnosis ideas and clinical management strategies.

## 2 Case report

**Figure 1:**
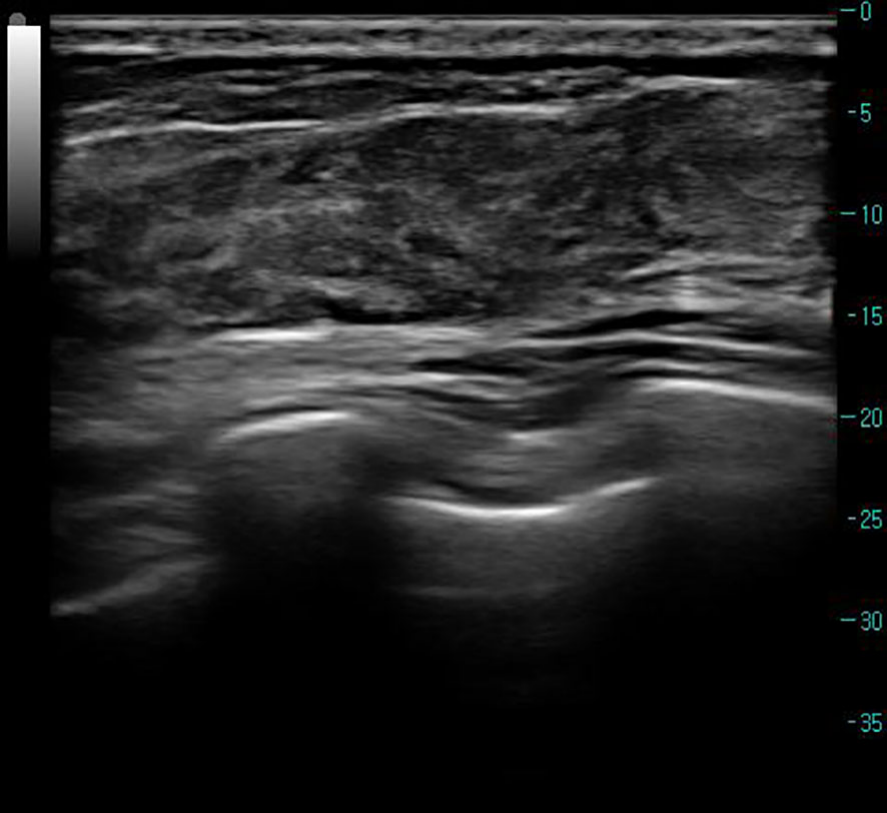
Ultrasound of left breast lesion. Preoperative ultrasound shows a hypoechoic nodule at the junction of the outer lower quadrant of the left breast and the areola, approximately 24 mm × 13 mm in size, with irregular shape, angular edges, uneven internal echo, and no blood flow detected. It is classified as BI-RADS 4a.

**Figure 2:**
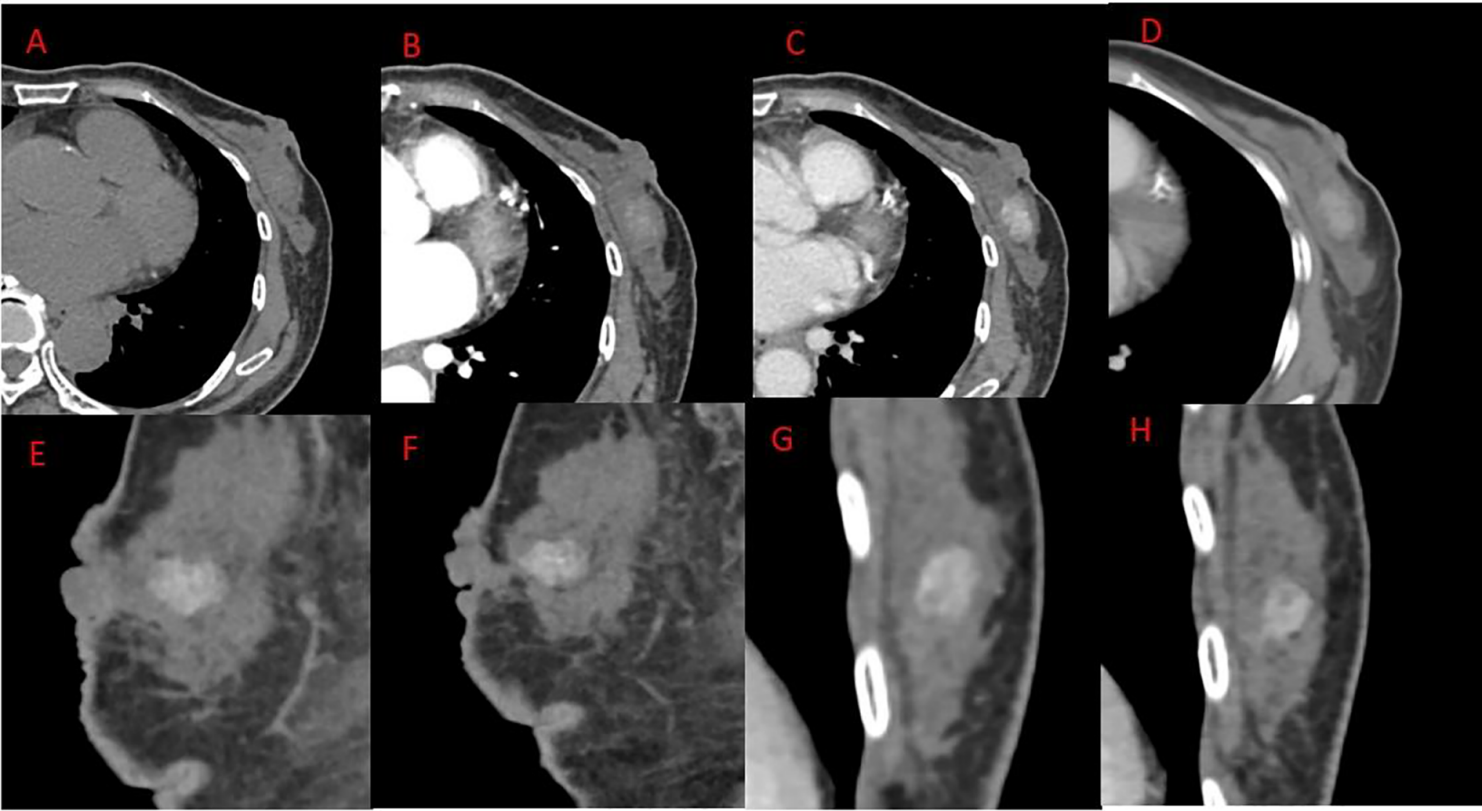
Chest CT plain + enhanced. **(A)** Plain scan phase, **(B)** arterial phase, **(C)** venous phase, **(D)** delayed phase, **(E, F)** venous phase sagittal, **(G, H)** venous phase. Coronal outside left breast first-order density soft tissue nodules were seen in the quadrant, 5 mm distant from the breast. An enhanced scan showed mild and uniform enhancement in the arterial phase and delayed enhancement in the venous and delayed phases. The edge of the lesion was unclear, and no calcification was found within it.

**Figure 3:**
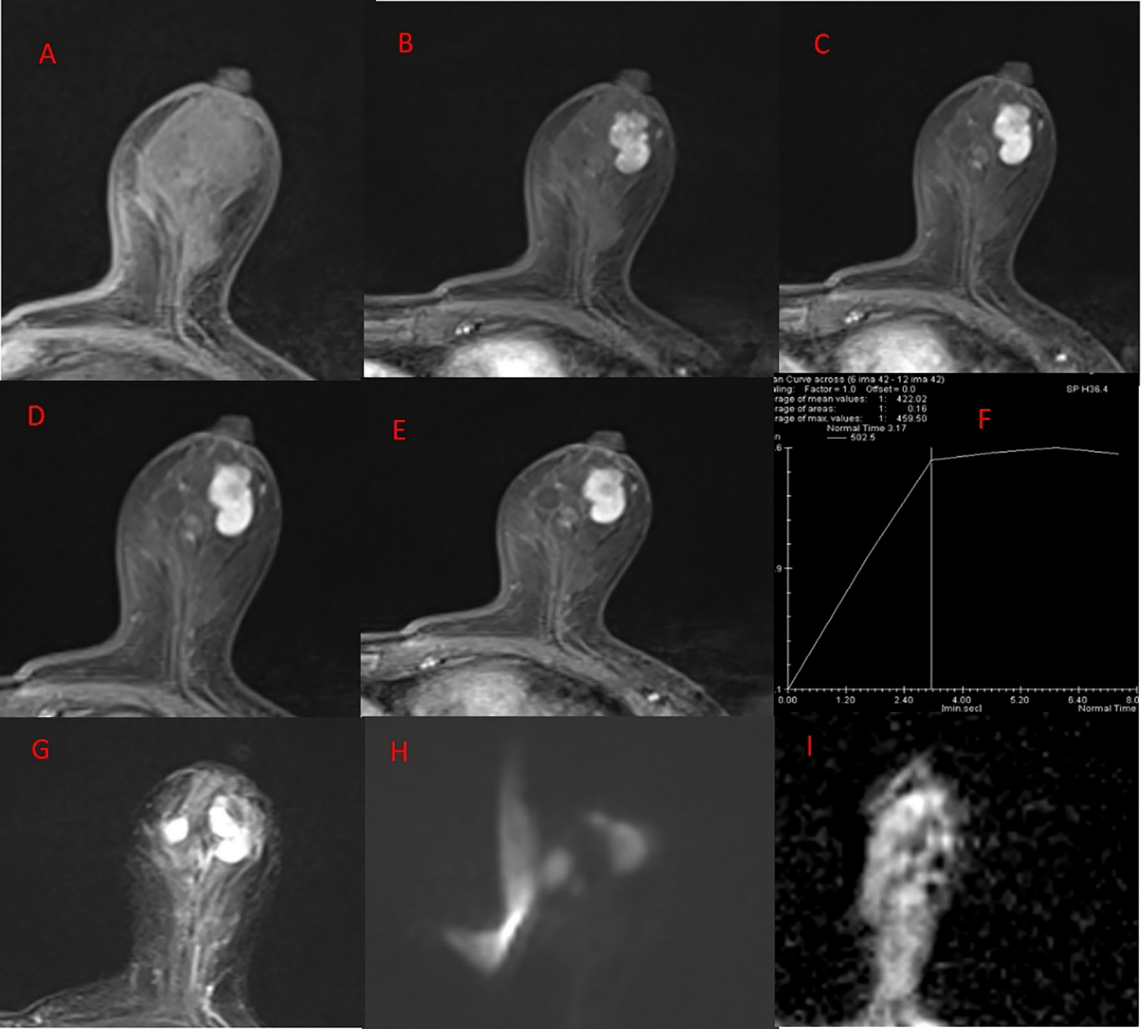
MRI plain scan + enhancement of left breast. **(A)** T1WI. **(B–E)** DCE at 1, 2, 4, 5, and 7 min, respectively. **(F)** TIC curve. **(G)** T2WI-FS. **(H)** DWI. **(I)** ADC. Left breast, outer inferior quadrant: The anterior view shows a nodular area with slightly longer T1 and T2 signal, lobulated shape, and with smooth edges and clear boundaries. DWI presents high signal intensity. ADC presents high signal intensity, with an ADC value of approximately 1.5 × 10^-3^. No significant diffusion restriction was observed. Dynamic enhancement scan shows obvious uniform enhancement, with an enhancement curve of inflow or plateau type.

**Figure 4:**
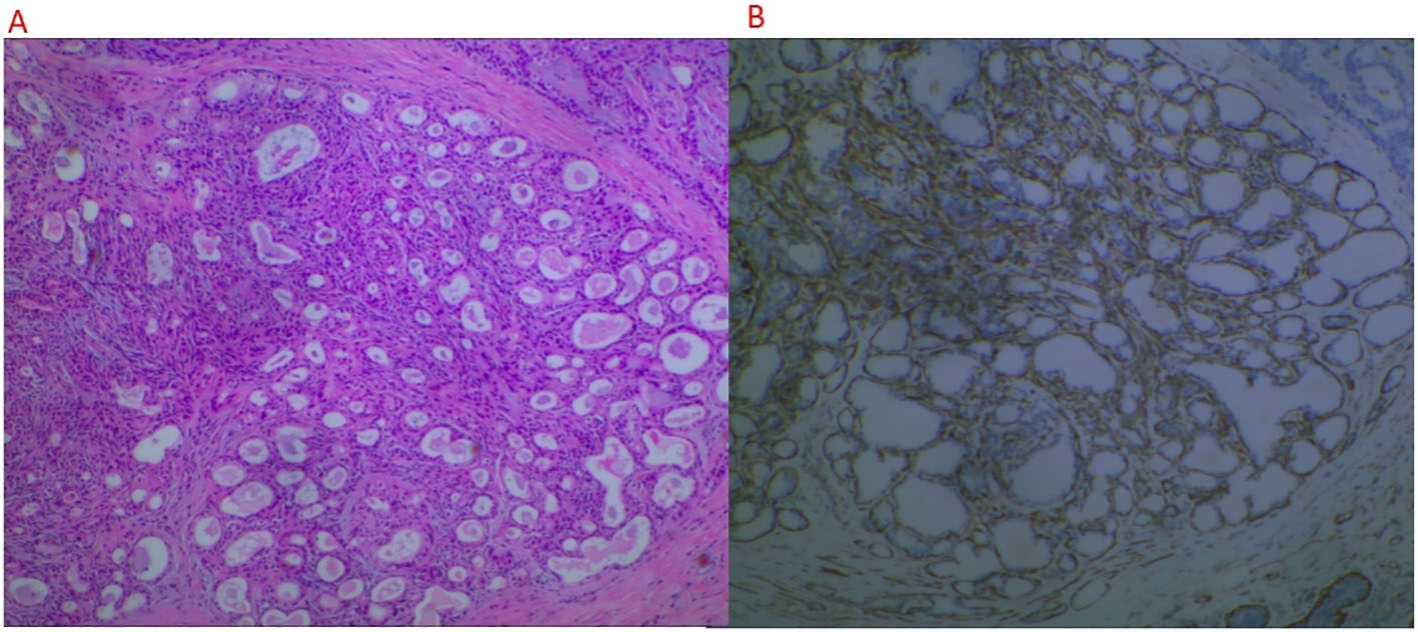
Pathological image. **(A)** Low-power microscopy (HE staining, 4 × 10). **(B)** Immunohistochemical staining (p63 positive). Microscopic examination: The tumor is composed of glandular epithelium and myoepithelial components, with glandular epithelial cells arranged in tubular, solid nests, or papillary structures, with some cells showing mild atypia. The myoepithelium is composed of spindle or stellate cells, distributed around the glandular epithelium. The stroma is mainly mucinous and chondromatous, with partial areas showing ossification. The lesion is consistent with salivary gland-like tumors, considered to be PA. The immunohistochemical expression is as follows: CK5/6 (+), P63 (basal cells+), SMA (myoepithelial+), CD117 (+), CK7 (+), and KI-67 (positive rate 5%).

The patient, a 66-year-old woman, with no family history of breast tumors, complained of a 2-day-old left breast tumor, about the size of a peanut, with no ulceration, pus, redness, swelling, or pain. Six months ago, the patient underwent a subtotal gastrectomy for gastric adenocarcinoma. Physical examination revealed a palpable mass in the outer lower quadrant of the left breast, approximately 20 to 30 mm in size, hard in consistency, with indistinct boundaries, irregular surface, fixed to underlying tissue, no obvious tenderness, and no local skin flushing or ulceration. No enlarged lymph nodes were palpable in the bilateral axillary and supraclavicular fossa. Ultrasound shows that, as shown in **Figure 1**, a hypoechoic nodule can be detected at the junction of the lower outer quadrant of the left breast and the areola, with a size of approximately 24 mm × 13 mm, irregular shape, angular edges, uneven internal echo, and no blood flow detected. It is classified as BI-RADS 4a. Contrast-enhanced CT showed an equal-density soft tissue nodule in the outer lower quadrant of the left breast as shown in **Figure 2**, 5 mm away from the nipple. The enhanced scan showed mild and uniform enhancement in the arterial phase and delayed enhancement in the venous phase and delayed phase, the edge of the lesion was unclear, and no calcification or cystic changes were found in the lesion. In the MRI plain scan + dynamic enhanced image in **Figure 3**, the lesion is shown to be isointense on the T1WI sequence; T2WI showed high signal with fat suppression, DWI showed high signal, and ADC showed high signal. The ADC value was about 1.5 × 10^-3^. There was no obvious diffusion restriction. The dynamic enhanced scan showed obvious uniform enhancement. The enhancement curve was inflow type or plateau type. The edge of the lesion was lobulated and the boundary was clear. The preoperative clinical and radiological preliminary diagnosis was a benign breast tumor, with a high possibility of fibroadenoma. The differential diagnosis also included phyllodes tumors, mucinous carcinomas, and metastases combined with the patient’s history of gastric cancer. Even though breast metastases are rare, they cannot be completely ruled out. Finally, the patient underwent breast lumpectomy, and pathology and immunohistochemistry confirmed pleomorphic adenoma of the breast (**Figure 4**). Due to the short postoperative period, long-term imaging follow-up data are still being collected for this case.

## 3 Diagnosis and differential diagnosis

### 3.1 Imaging diagnostic analysis

Based on the multimodal imaging findings of this case, we have extracted the following key features:

1. Morphology: The ultrasound shows irregular shape and angular edges, while MRI shows clear boundaries and lobulated edges, suggesting that the tumor has a certain degree of atypicality but overall shows an expansive growth pattern (7). The ultrasound often shows ill-defined lesion margins due to interference from surrounding glandular tissue, echo attenuation, and interface reflections. In contrast, T2WI fat-saturation sequence of MRI clearly revealed the high signal intensity of the mucinous stroma, thereby more accurately delineating the lobulated margins and expansile growth pattern of pleomorphic adenomas.
2. Signal/density characteristics: The plain CT scan shows isodense findings, while the T2WI fat-saturation sequence of MRI shows a significant high signal; this feature is particularly prominent.
3. Hemodynamics: CT and MRI dynamic enhancement scans show progressive and uniform enhancement, with time–signal intensity curves (TIC) as “inflow type”.
4. Diffusion characteristics: DWI shows a high signal, but the ADC map signal is also increased, with ADC values of about 1.5 × 10^-3^, suggesting no diffusion restriction.

### 3.2 Differential diagnosis

Based on the patient’s age and history of gastric cancer, it needs to be differentiated from the following lesions:

1. Fibroadenoma: Common benign tumor, but the typical T2WI signal is often slightly low or isodense, and there are often septa (9), rarely showing the significant high signal as in this case, and its shape is usually more regular.
2. Breast metastasis: Combined with the history of GC, this is an important differential point. The core of differentiation that lies in the T2WI of metastases is often of moderate to low signal, and usually there is diffusion restriction usually presenting as ring enhancement (10, 11), which is completely different from the imaging features of this case. In addition, there is no axillary lymph node enlargement in this case, which also supports it as a primary lesion.
3. Phyllode tumor of the breast: This is a greater challenge for differentiation, as it can also show as T2WI high signal and lobulated, but lobular tumors are usually larger in size, grow faster, and are prone to cystic degeneration or necrosis internally, with more significant and uneven enhancement, which is different from the uniform enhancement in this case (12).
4. Mucinous carcinoma: This is also characterized by significant T2WI high signal. The key difference lies in the dense cell areas of mucinous carcinoma causing diffusion restriction, and the enhancement is often characteristically “peripheral enhancement” or uneven enhancement (13), which is inconsistent with the findings in this case. The breast tumor in this case was pathologically confirmed as PA without malignant features; thus, no further genetic testing was performed. However, in clinical practice, patients with concurrent gastric and breast cancers (especially invasive lobular carcinoma) should be evaluated for hereditary syndromes such as hereditary diffuse gastric cancer (HDGC). Screening for CDH1 gene mutations and genetic counseling are recommended.
5. Lymphoma/leukemia breast involvement: This often presents as a palpable, painful mass, frequently multiple, commonly located in the upper outer quadrant, accompanied by axillary lymph node enlargement. On mammography, it typically appears as a poorly defined mass; ultrasound may show it as hyperechoic or hypoechoic with heterogeneous echogenicity. MRI commonly reveals an irregularly shaped mass with mild heterogeneous enhancement, TIC as “outflow type”, and restricted diffusion (10).

Preoperative comprehensive imaging evaluation, especially the characteristic T2WI significant high signal, no diffusion restriction, and benign enhancement pattern of MRI, strongly suggests a benign tumor rich in mucinous matrix, and pleomorphic adenoma should be considered, but it is difficult to completely exclude lobular tumor. The final diagnosis depends on pathological confirmation.

## 4 Discussion

PA usually occur in the salivary glands and rarely in the breast. PA of the breast was first reported in 1906 (14). PA of the breast are pathologically characterized by a mixture of epithelial/myoepithelial components and myxoid stroma/chondroid components (15). PA of the salivary glands and pleomorphic adenomas of the breast share many of the same pathological and MRI imaging features, such as clear borders, obvious high signal on T2WI, and no obvious diffusion restriction. These features are considered to be related to the rich myxoid stroma (13). This patient showed obvious high signal on T2WI, which is also because the stroma of the lesion is dominated by myxoid and chondroid stroma, and this is also one of the important points of differentiation from fibroadenoma, which is common in the breast. To date, fewer than 100 cases of pleomorphic adenoma of the breast have been reported, with even fewer imaging studies available. **Table 1** shows a list of selected cases describing partial imaging features (primarily ultrasound) of pleomorphic adenoma of the breast.

**Table 1:** This case and previously reported cases of the imaging features of PA of the breast.

Case	Present case	Ito et al., 2022 (MRI case) (4)	Ginter et al., 2015 (8)	Ahmad et al., 2023 (16)	Teh et al., 2021 (6)
Age/sex	66/woman	43/woman	42/woman	66/woman	63/woman
Clinical history	Palpable mass in the left breast for 2 days; history of gastric cancer surgery	Left breast mass slowly enlarging over 20 years	Slowly growing palpable mass in the left breast	Several-month history of a breast mass	Asymptomatic lesion detected on breast cancer screening
Location	Lower outer quadrant of the left breast, 0.5 cm from the nipple, subareolar region	Upper outer quadrant of the left breast, close to the nipple	Retroareolar region, 2 cm from the nipple	Periareolar region	3 o’clock position of the left breast, 6 cm from the nipple
Maximum size (cm)	2.4	5	2 to 3	5.5	2 to 3
US	An ill-defined, hypoechoic, irregular mass with a heterogeneous internal echotexture and no detectable vascularity	A well-circumscribed hypoechoic mass with multiple cystic areas and internal vascularity	Fibroadenoma-like or complex cystic-solid mass	Hypoechoic, lobulated mass with coarse calcifications	Relatively well-defined mass with heterogeneous echotexture and small calcifications; no detectable vascularity
CT	An iso-dense nodule with progressive, homogeneous enhancement and slightly indistinct margins. There is no evidence of calcification or cystic change	N/A	N/A	N/A	N/A
MRI T1WI/T2WI signal	Isointense on T1WI, markedly hyperintense on T2WI-FS with well-defined, lobulated margins	Solid component: low T1, high T2/STIR signal; cystic component: slightly high T1 and slightly low STIR signal	N/A	N/A	N/A
Follow-up	Short follow-up; no recurrence detected so far	No recurrence at 1-year follow-up	No recurrence at 6-month follow-up	N/A	N/A
Enhancement pattern (CT/MRI) and DWI	Dynamic CT and MRI show homogeneous progressive enhancement; TIC shows inflow/plateau type; no diffusion restriction	DCE-MRI: solid component shows fast/plateau enhancement pattern; cystic areas show no enhancement, no diffusion restriction	N/A	N/A	N/A
Pathology/immunology	Epithelial + myoepithelial biphasic components, mucinous/cartilaginous matrix with focal ossification; CK5/6, p63, SMA, CD117, CK7 positive, Ki-67 approximately 5% no malignancy	Epithelial/myoepithelial hyperplasia, rich in mucinous matrix; no malignant components	Stromal (bland spindle cells embedded in a myxochondroid) + epithelial	Cartilage components ER/PR (<1% cells) HER2/neu (0), Ki-67 approximately 3% CK5/6, p63 positive	Epithelial + myoepithelial + mucinous/cartilaginous matrix

Shuichi Ito et al. (4) once reported a case of MRI manifestations of breast pleomorphic adenoma. It was an oval mass in the left breast with smooth borders. It was composed of solid and cystic components. There was no diffusion restriction. The TIC of the solid part was inflow or plateau type. The difference in this case was that the lesion was smaller and no cystic part was found. This case demonstrates combined features from ultrasound, contrast-enhanced CT, DCE-MRI, and DWI/ADC. Compared to most literature reporting only a single imaging modality, it more comprehensively reflects the imaging–pathology correlation—specifically the high T2 signal and absence of diffusion restriction—resulting from the abundant mucinous/cartilaginous matrix in PA of the breast. Secondly, unlike previous cases, this patient had a history of GC. Therefore, we must differentiate this from breast metastases. Breast metastases are mainly treated with adjuvant therapy, and the treatment effect is poor, the prognosis is poor, and the survival period is short (17). Primary extramammary tumors are mainly gastric cancer and lung cancer in Asia, while melanoma, lung cancer, and ovarian cancer are the main tumors in Europe and America. Leukemia and lymphoma are also common primary tumors (18). MRI appears like benign tumors, with smooth edges, no burrs, and lobulation, but diffusion is often limited, T2WI signal is low, TIC curves are mostly flat or outflow type, often accompanied by axillary lymph node enlargement, and the lymphatic hilum is unclear (10). In this case, the T2WI signal was obviously high and there was no obvious diffusion restriction. The axillary lymph nodes were not enlarged. The imaging findings are inconsistent with metastatic lesions. With advances in genomics technology, next-generation sequencing (NGS) performed on tissue biopsies or liquid biopsies can further aid in differential diagnosis. Pleomorphic adenomas may carry characteristic gene rearrangements (e.g., PLAG1 or HMGA2), while primary breast cancers or metastatic tumors typically exhibit distinct mutation profiles, such as TP53 mutations (common in triple-negative and high-grade cancers) and PIK3CA hotspot mutations (e.g., E542K, E545K, H1047R, present in approximately 30%–40% of HR+/HER2− breast cancers) (19, 20). When tissue sampling is limited, plasma ctDNA analysis can also identify systemic malignancies. Although NGS testing was not performed in this case due to a pathologically confirmed benign diagnosis, recognizing the potential value of these genomic tools is particularly important in patients with a history of other malignancies. This can help guide treatment decisions and avoid overtreatment.

## 5 Conclusion

We report a case of PA of the breast with the goal of improving people’s understanding of its imaging characteristics, which is characterized by a significantly high signal on T2WI and no diffusion restriction on MRI. For patients with a history of malignant tumors, recognizing these characteristics can help distinguish them from breast metastases and other breast tumors, providing the correct diagnostic direction for the clinic, thereby avoiding over-treatment and misdiagnosis for patients. However, the final diagnosis depends on pathological confirmation.

## Data Availability

The original contributions presented in the study are included in the article/supplementary material. Further inquiries can be directed to the corresponding author.
